# Supplementary material for: Intranasal Mitochondrial Transplantation Restores Mitochondrial Function and Modulates Glial–Neuronal Interactions in a Genetic Parkinson’s Disease Model of UQCRC1 Mutation
Source: Cells. 2025 Jul 25;14(15):1148. doi: 10.3390/cells14151148 (PMC12346496; doi:10.3390/cells14151148)

Supplementary Figure

Supplementary Figure S1. CsA preloading and mitochondrial internalization efficiency. (A) Spectrophotometric analysis of CsA absorbance at 210 nm was performed to determine the peak CsA concentration used for mitochondrial preloading (upper panel). The CsA standard curve was established using serial dilutions of CsA concentrations (lower panel) for the quantification of mitochondrial CsA accumulation. (B) Absorbance values of mitochondria incubated with varying CsA concentrations (1, 3, 5, and 10 μg/μL) were measured. Data were presented as means ± SD. N=3 per group. (C) Internalization efficiency (percentage) of mitochondria without (MT) and with CsA accumulation (MT-CsA) was assessed by tracking mitochondrial prelabeling with MitoTracker Red staining in cybrid cells harboring MERRF mitochondrial DNA mutations. CsA modification did not alter mitochondrial uptake across different doses.

Figure S1

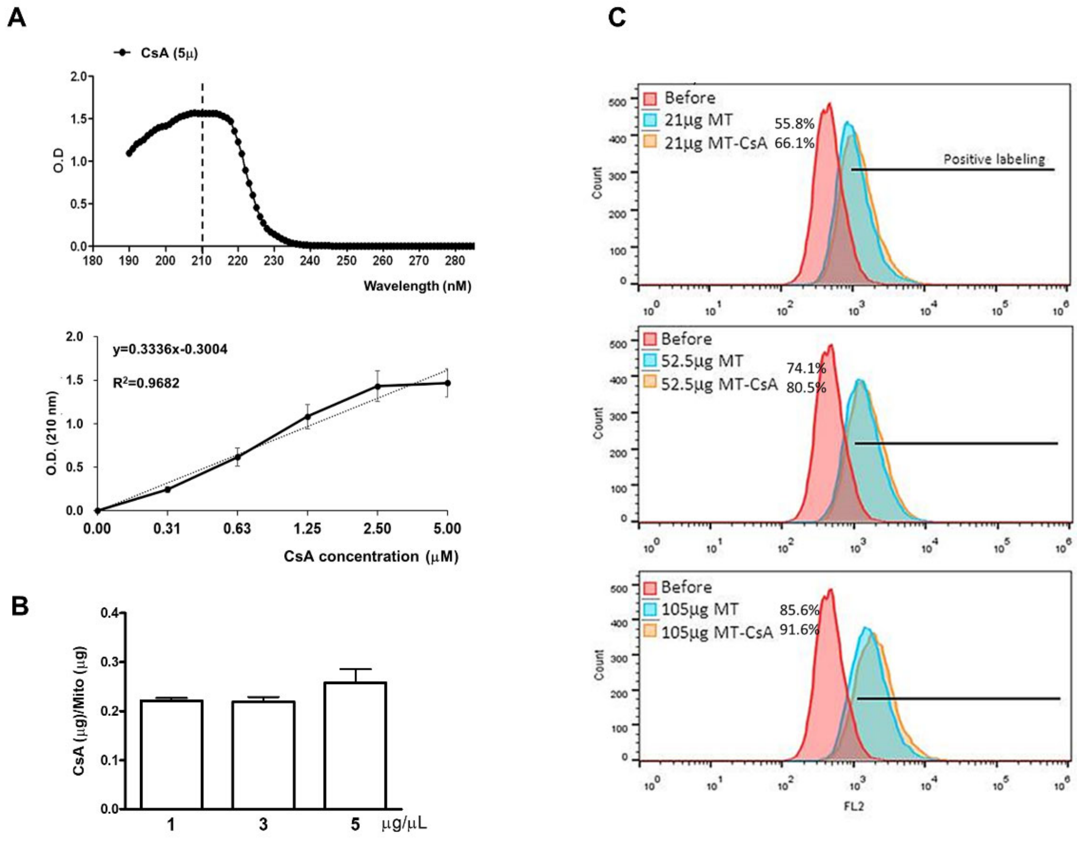

Supplementary Figure S2. Effect of intranasal cyclosporin A (CsA) administration on locomotor function and mitochondrial complex III (CIII) activity in substantia nigra (SN) neurons of *UQCRC1* mutation (p.Tyr314Ser) knock-in mice. (A) Open-field test results showing total movement distance (cm), movement duration (s), velocity (cm/s), and zone frequency (number of entries into specific areas) in *UQCRC1* mutant mice treated with intranasal administration of CsA alone (UQ-CsA) or untreated (UQ). CsA was administered at a dose of 10.31 ± 1.101 μg per mouse per week for six months, equivalent to the CsA concentration accumulated in mitochondria when 40 μg of mitochondria was transplanted per week. (B) The CIII enzymatic activity in SN of UQCRC1 mutant mice was analyzed after CsA-alone treatment for 6 months. Data were presented as means ± SEM. *P < 0.05 compared with the wild-type (WT) control. N = 6 per group.

Figure S2

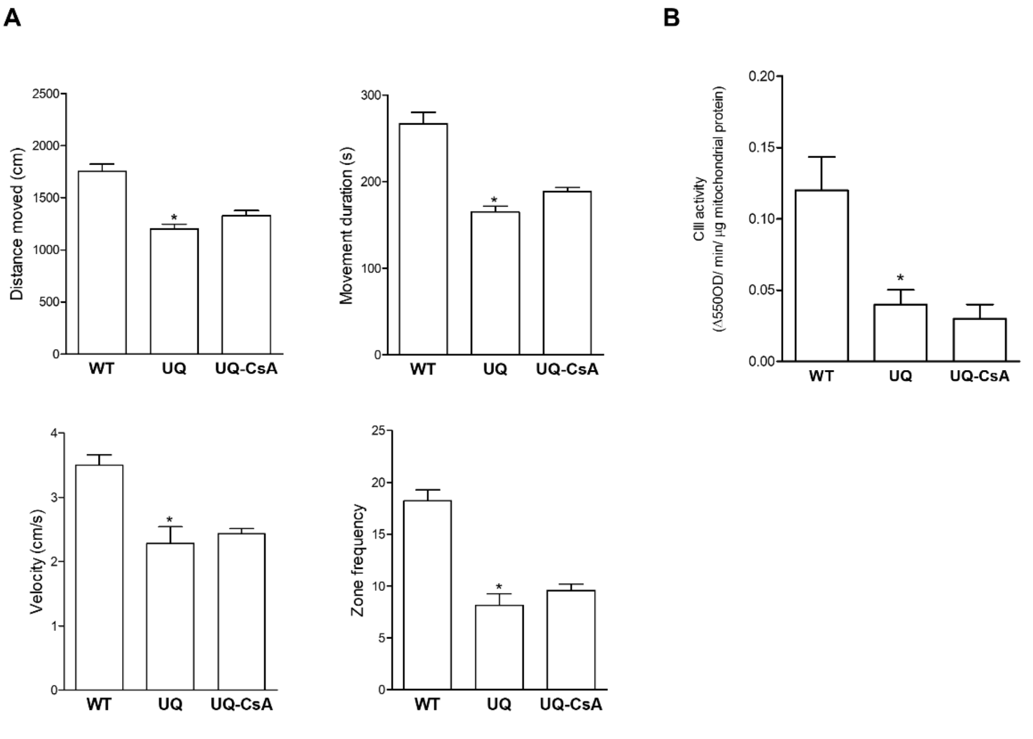

Supplement: Supplementary file 1 [file cells-14-01148-s001.zip › cells-3699437-supplementary.pdf]
